# Proposing a Patellar Tendinopathy Screening tool following a systematic review

**DOI:** 10.4102/sajp.v74i1.454

**Published:** 2018-09-26

**Authors:** Sanell Morgan, Frederik F. Coetzee

**Affiliations:** 1 Department of Physiotherapy, University of the Free State, South Africa; 2 Department of Exercise and Sport Sciences, University of the Free State, South Africa

## Abstract

**Background:**

Patellar tendinopathy (PT) is an overuse injury of the knee. The mechanism of injury is associated with repetitive stress on the patellar tendon of the knee as a result of explosive movement. Patellar tendinopathy is prevalent in all populations and is associated with intrinsic and extrinsic risk factors.

**Objectives:**

Primarily, the objective was to report on the intrinsic and extrinsic risk factors for PT, entailing a systematic review of the literature; the secondary objective was to use these risk factors to compile a proposed PT screening tool from the review and standard outcome measures.

**Method:**

A systematic review was undertaken according to the Preferred Reporting Items for Systematic Reviews and Meta-Analyses (PRISMA) guidelines. Elimination criteria of the articles included duplicates, titles, abstracts and methodological quality. The evidence was collected, characterised with regard to the intrinsic and extrinsic risk factors and summarised descriptively.

**Results:**

The search yielded 157 feasible articles prior to commencement of article elimination. Six articles were included with a mean methodological quality score of 69%. Eight intrinsic and five extrinsic risk factors were identified. These identified risk factors are all relevant to the pathology and formed the basis for a proposed PT screening tool. The Victorian Institute of Sports Assessment for Patellar Tendinopathy Questionnaire, Visual Analog Scale and the Pain Provocation Test are also included in the proposed test.

**Conclusion:**

Intrinsic and extrinsic risk factors for PT were identified, and consequently, the proposed PT screening tool was formulated for possible future testing in appropriate studies.

**Clinical implications:**

Prevention of PT through intrinsic and extrinsic risk factor identification, and implementation in the clinical setup as a possible outcome measurement tool with which to verify functional improvement in PT rehabilitation.

## Introduction

Patellar tendinopathy (PT), an overuse injury (Reinking 2016) often referred to as ‘jumper’s knee’, commonly occurs in sports that particularly involve jumping. The mechanism of injury is associated with continuous and repetitive stress on the patellar tendon of the knee (Celebi et al. 2016) resulting from explosive movement (Van Ark et al. 2016). This pathology occurs in all populations (Celebi et al. 2016), with an estimated prevalence of 8.5% in recreational athletes who usually have a less intense training regimen (Vetrano et al. 2013). However, in elite athletes the prevalence of PT is as high as 13% – 20% (Stuhlman et al. 2016) and can even reach a percentage of 40% in elite athletes involved in sports with a high demand (Abat & Sanchez-Ibañez 2014) on the extensor mechanism of the knee (Steunebrink et al. 2013). The main causes are intrinsic (Malliaras & O’Neill 2017) and extrinsic risk factors (Hägglund, Zwerver & Ekstrand 2011; Reinking 2016).

The physical diagnosis of PT is based on clinical and predominantly ultrasound examination, although findings may not necessarily be associated with the severity of the symptoms (Celebi et al. 2016). The rehabilitation of PT is typically non-operative and requires an evidence-based approach founded on the research literature. This is enhanced by the clinical judgement of the rehabilitation team, their understanding of the rehabilitation intervention and wise implementation thereof (Rutland et al. 2010), whilst still keeping the athlete’s return to sport goals in mind (Reinking 2016). Patellar tendinopathy rehabilitation is an encompassing management of the athlete with the fundamental focus on exercise (Van Ark et al. 2016). However, with the possibility of enduring persistent symptoms, full recovery from this pathology is not always certain (De Vries et al. 2017).

Even though PT is a familiar pathology amongst elite and recreational athlete populations (Reinking 2016), there is modest evidence for risk screening tools in PT assessment and rehabilitation. A necessity is that the rehabilitation team’s clinical reasoning and progress of the rehabilitation process must be founded on authenticated subjective and objective measurements (Reinking 2016). As a result of the unconvincing (Peters et al. 2016) and challenging track record of PT rehabilitation (Rodriguez-Merchan 2013; Van Der Worp et al. 2011b), compelling and trustworthy screening tools are important for routine clinical practice. These should be used as outcomes to validate the efficiency of the treatment, monitor progress and identify regression of the pathology (Celebi et al. 2016).

Prior systematic reviews have identified a list of risk factors for PT, but there is a paucity of information on PT screening tools for practical implementation in the clinical setup. Therefore, a need was identified for an in-depth systematic literature review to form the basis of a draft screening tool for PT as well as updating current data in this field. Such a PT screening tool may facilitate PT research, once it has been appropriately developed. Clinically, it may then assist in prevention of PT by early identification of risk factors and serve as an outcome measure in rehabilitation. Thus, the primary objective of this study was to systematically identify and appraise studies reporting on the intrinsic and extrinsic risk factors for PT, with the secondary aim of using the identified risk factors in a proposed PT screening tool, in combination with appropriate outcome measures.

### Method

Systematic reviews are a cornerstone in research for complete, accurate and reliable recapitulating of evidence, and consequently provide several benefits to clinicians (Liberati et al. 2009). This systematic review was undertaken according to the Preferred Reporting Items for Systematic Reviews and Meta-Analyses (PRISMA) guidelines (Moher et al. 2009). The inclusion and exclusion criteria in Box 1 were used as a basis for the consideration of articles.

Box 1 Inclusion and exclusion criteria.

**Inclusion criteria**

**Publication period:** Published articles dated January 2010 to September 2017.

**Research design:** Quantitative research studies, randomised clinical trials, non-randomised clinical trials and systematic reviews.

**Participants’ age:** 18–60 years.

**Population:** Sporting and general.

**Research focus:** Identification of the intrinsic and extrinsic risk factors to allow the formulation of a draft Patellar Tendinopathy Screening tool.

**Exclusion criteria**

**Population:** Participants with previous knee surgery, knee injection therapy or other knee pathologies.

**Research focus:** Articles not related to the intrinsic and extrinsic risk factors for patellar tendinopathy.

**Article language:** Non-English articles

**Article availability:** Articles for which only an abstract was available.

### Search strategy

The authors conducted two independent systematic review searches, first in October 2017 and in January 2018, for articles published between January 2010 and September 2017, with an allotted time period (approximately three months apart) between the searches by the authors. This ensured that results cross-referenced and that all eligible articles were included in the review. This interval was chosen to be a supplementary addition to a previously published systematic review on the causative risk factors and rehabilitation for PT in which articles were selected only up to October 2015 (Morgan, Janse van Vuuren & Coetzee 2016). *Clinical Sports Medicine* (5th edition) by Brukner and Khan (2017) was used as a basis to identify the words for the Boolean phrases.

EBSCO host electronic databases were searched and included the following: Africa-Wide Information, ERIC, Academic Search Complete, AHFS Consumer Medication Information, CINAHL with Full Text, MEDLINE with Full Text, Health Source: Nursing/Academic Edition, Health Source – Consumer Edition, PsycARTICLES, Humanities Source, PsycEXTRA, SocINDEX with Full Text, PsycINFO, PsycTESTS, and SPORTDiscus with Full Text.

The following keywords were applied in the search strategy for the identification of applicable articles for the review:

(‘patella* tendinopath*’ or [patella* and tendinit*])(‘intrinsic factor*’ or age or gender or ‘body composition*’ or ‘fat mass’ or ‘body weight’ or ‘body mass index’ or injur* or ‘joint instability*’ or ‘musc* strength’ or ‘musc* power’ or ‘range of motion’ or ‘range of movement’ or ‘anatomic* alignment*’ or ‘postural stability*’ or ‘sport* specific technique*’ or ‘level of skill*’ or ‘skill* level*’ or ‘extrinsic factor*’ or strapping or bracing or ‘foot wear’ or footwear* or shoe* or ‘training surface*’ or ‘eccentric decline squat*’ or ‘skill* acquisition’ or proprioception* or flexib* or ‘muscle activat*’ or etiolog* or aetiolog*) AND (rehab* or ‘return to sport’ or ‘return to play’ or ‘motor re-educat*’) and (exercise* or train* or sport*)

### Study selection

**FIGURE 1:**
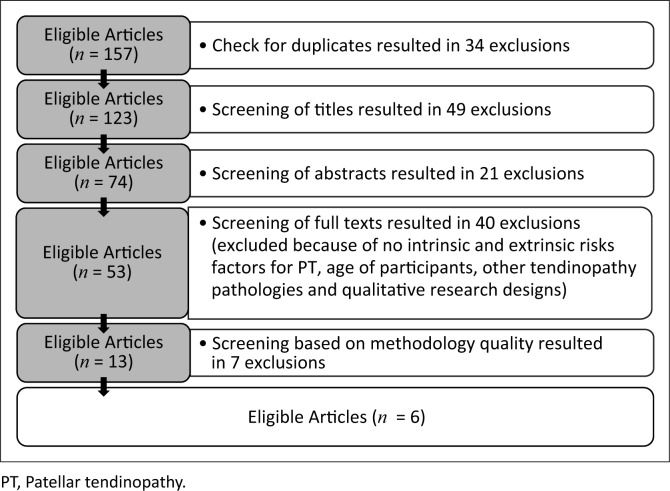
Flow diagram of search strategy to determine the final sample of articles for the systematic review.

Each author undertook the study selection process according to the inclusion and exclusion criteria in Box 1. All uncertainties regarding the eligibility of articles were discussed by the authors to clarify and agree upon any initial discrepancy. Figure 1 demonstrates the methodology of the search strategy in determining the final set of articles for evaluation.

### Methodological quality of the articles included in the review

The eligibility and quality of the articles were appraised by the authors using two checklists (Table 1). More than one checklist was needed because of the dissimilar research designs described in the different articles. These specific checklists were selected to assist in defining the methodological quality of each article because of their good validity and reliability in the literature (National Institute for Health and Excellence [NICE] 2012; Shea et al. 2009; Winn 2013). The checklists included the AMSTAR (Shea et al. 2009) and NICE (2012) *Methods for the Development of NICE Public Health Guidance* checklists. The individual methodology scoring for each article is displayed in Appendix 1.

**TABLE 1:** Methodological assessment of included articles.

Study design	Methodological checklist	Range of quality scoring	Quality scoring average	Article exclusion reason
Systematic reviews	AMSTAR checklist	8/11 (*n* = 1)	73% (*n* = 1)	Methodology scoring of 50% or less
Quantitative research	National Institute for Health and Excellence checklist	15/27 – 22/27 (*n* = 5)	67% (*n* = 6)	Methodology scoring of 50% or less

The methodological quality scoring was performed on 13 articles that met the inclusion criteria, with 6 articles remaining for the systematic review.

### Data extraction

The data contained in the eligible articles were extracted and incorporated into a customised Microsoft Excel data spread- sheet developed by the authors (Tables 2 and 3). The documented information included authors, year of publication, aim of the study, type of study, the population, level of participation and intrinsic and extrinsic risk factors defined in each article. The intrinsic and extrinsic risk factors were identified in the results and discussion of each article during the data extraction process. The authors conducted the data extraction individually, which was followed by a final mutual check.

**TABLE 2:** Characteristics of the included articles.

Authors and year of publication	Aim of the study	Study design	Study population	Level of participation
De Vries et al. (2015a)	Investigating if the identified risk factors in a previous study in 2008 can prospectively be identified as risk factors of PT in 2011	Survey-based prospective cohort study	Male and female	Not applicable
Mendonça et al. (2016)	Determine the connotation of lower limb muscle strength and lower limb alignment, range of motion or flexibility in male athletes with PT	Cross-sectional study	Male	Elite athletes
Van Der Worp et al. (2011b)	Identification of the risk factors for PT	Systematic review	Male and female	Elite and recreational athletes
Toppi et al. (2015)	Determining the prevalence of magnetic resonance imaging–diagnosed PT in middle-age community-based women and the factors associated	Prospective cohort study	Female	General population
Van der Worp et al. (2011a)	Work-related etiological factor identification for PT and the connection amongst work limitations and PT	Online survey: descriptive	Male and female	Elite and recreational athletes
Van der Worp et al. (2012)	Identification of risk factors of PT in volleyball and basketball players	Cross-sectional study	Male and female	Elite and recreational athletes

PT, Patellar tendinopathy.

**TABLE 3:** Intrinsic and extrinsic risk factors for patellar tendinopathy.

Author cited the risk factor	Intrinsic risk factor	Extrinsic risk factor	Risk factor ratio or prevalence	*p*
De Vries et al. (2015a)	Male > female	Hard physical work in combination with jumping sports	Not applicable	Not applicable
Mendonça et al. (2016)	Decreased ITB flexibility Shank forefoot alignment	Not applicable	Not applicable	Decreased ITB flexibility *p* = 0.006 Shank forefoot alignment *p* = 0.013
Toppi et al. (2015)	Increased Vastus Medialis size or muscle strength	Physical activity	Prevalence of MRI defined patellar tendinopathy for increased Vastus Medialis size or strength and physical activity was 30.1%	MRI defined patellar tendinopathy Cross-sectional area of Vastus Medialis (cm^2^) Univariate analysis *p*-value = 0.03 Multivariate analysis *p* = 0.02 Physical activity Univariate analysis *p* = 0.03 Multivariate analysis *p* = 0.02
Van der Worp et al. (2011a)	Male > female	Heavy physical demanding work in volleyball and basketball players	Twice as high in men in relation to women (24.8% vs. 11.9%)	Not applicable
Van Der Worp et al. (2011b)	Weight, body mass index, waist-to-hip ratio, leg length variance, arch height of the foot Decreased flexibility in the quadriceps and hamstring muscle, decreased quadriceps muscle strength, vertical jump	Not applicable	Not applicable	Not applicable
Van der Worp et al. (2012)	Male > female Increased age (no specific conclusion regarding a specific age group)	Level of sports participation (elite vs recreational) Type of sport: Jumping sports (volleyball)	Prevalence male > female (25.3% vs 13.1%)	Male > female (*p* < 0.001)

ITB, Iliotibial band; MRI, magnetic resonance imaging.

### Analysis of data

Combining of the data for the formulation of a meta-analysis was not the intention of this systematic review because of the differences of the results in terms of the variety of articles with different study populations. All the empirical evidence was collected, characterised with regard to the intrinsic and extrinsic risk factors for PT and summarised descriptively.

### Ethical considerations

Ethics approval was obtained from the Ethics Committee of the Faculty of Health Sciences, University of the Free State (approval no. 181/2015).

## Results

The collective results of both independent searches yielded 157 feasible articles for inclusion prior to the commencement of article elimination (Figure 1 and Table 1). Six articles met all the inclusion criteria and are shown in Table 2. These were included in the systematic review with a mean methodological quality score of 69% (Table 1). Only quantitative research designs and systematic reviews formed part of the included articles with, surprisingly, no randomised clinical trials. This systematic review led to the identification of eight intrinsic and five extrinsic risk factors, with the results of these articles being discussed.

The demographic information obtained from the systematic review shows that all six of the included articles described the study population. Two articles consisted of exclusively either male or female participants, whilst the other four articles described both male and female participants. Eighty-three per cent (*n* = 5) of the included articles provided detail on the level of participation. A combination of elite (66%) and recreational participants (50%) was described in four articles, with one article having a general study participant population.

Eight intrinsic risk factors associated with PT were identified in the articles that described their intrinsic risk factors. Categorisation of these risk factors included three main intrinsic risk factors, namely gender (male > female), impaired lower limb muscle flexibility and muscle strength. The other identified intrinsic risk factors were body composition, leg length variances, anatomy of the foot, lower patellar pole and age of the study participants.

Five extrinsic risk factors for PT were identified with the main extrinsic risk factor being the common prevalence of PT in sports that involve jumping (50%). The additional four extrinsic risk factors were heavy physical work in combination with jumping sports, level of sport participation (elite vs recreational level), physical activity and type of sport.

## Discussion

PT is a well-recognised pathology with an inclusive aetiology of intrinsic non-modifiable and intrinsic and extrinsic modifiable risk factors that are directly linked to overloading of the patellar tendon (Hägglund et al. 2011). It is still unclear and debateable as to which particular mechanisms might be responsible for influencing intrinsic systemic factors for the risk of developing PT (Malliaras & O’Neill 2017). According to the outcomes in this systematic review, gender (Morton et al. 2017) and age were non-modifiable intrinsic risk factors for PT. Gender was found to be a statistically similar finding to that reported by De Vries et al. (2015a), Van Der Worp et al. (2011b) and Van der Worp et al. (2012) that men are more likely to develop PT. This finding is also similar to results reported by Rudavsky and Cook (2014), indicating that male participants have a greater risk of sustaining PT than female participants. It is believed that oestrogen in female participants may act as a protective mechanism and this may explain the discrepancy between genders, although this needs further investigation (Torres, Zgonis & Bernstein 2013).

The second non-modifiable intrinsic risk factor was increased age, granted that there was no conclusion regarding a specific age group in the results. The evidence shows that age may be associated with altered cellular activity in the patellar tendon, muscular function and mechanical properties (Malliaras & O’Neill 2017) and these age-related changes increase the predisposition of the general population to develop PT (Murtaugh & Ihm 2013).

In general, the anatomy and biomechanics of each individual athlete or person have an influence on the risk of developing PT (Malliaras & O’Neill 2017). Therefore, different morphologies of the foot (Reinking 2016), place increased strain and load on the patellar tendon (Schwartz, Watson & Hutchinson 2015). In terms of leg length variance, the longer leg is generally associated with PT pathology with no other specific explanation (Van der Worp et al. 2011b) as to whether this is an anatomical or functional discrepancy. A lower patellar pole might be explained by the impingement theory, which implies the irritation of the patellar tendon on different knee flexion degrees (Torres et al. 2013). These three intrinsic risk factors for PT might be modified and improved in rehabilitation, but this has not been demonstrated in studies. One possible option in the rehabilitation of PT is the use of strapping or orthosis, which may potentially have a positive effect (De Vries et al. 2015b).

There are a further eight modifiable risk factors for PT which can be either intrinsic or extrinsic. Impaired quadriceps, hamstring and iliotibial band muscle flexibility is an intrinsic risk factor for PT (Malliaras & O’Neill 2017; Reinking 2016; Schwartz et al. 2015). Muscle stiffness is related to general overuse of muscles, especially muscles in the lower limb and knee region (Brockmeyer et al. 2015) and injured athletes constantly present variations in flexibility deficits (O’Sullivan, McAuliffe & Deburca 2012). As highlighted by Carvalho et al. (2012) and Rogan et al. (2013), lower limb stretching in athletes is necessary for optimal performance in sport, injury prevention and rehabilitation. Improving flexibility of the lower limbs has always been a focus area in the rehabilitation of PT (Rudvasky & Cook 2015) and special attention is paid to improving the flexibility of the quadriceps femoris, hamstring and heel cord muscles by stretching programmes (Vetrano et al. 2013). This component of rehabilitation may benefit faster return to sport (O’Sullivan et al. 2012) by minimising symptoms (Rutland et al. 2010).

Impaired muscle strength of the quadriceps femoris increases the likelihood of developing PT (Rudvasky & Cook 2015). The rationale behind this is that lower leg muscle strength, especially weakness surrounding the knee joint, contributes to patellar tendon strain by the abnormal distribution of load and malalignment of patellar tracking (Torres et al. 2013). To address this, it is advised that when strengthening is commenced during rehabilitation, one should start with isometric strengthening of the quadriceps muscle because of its pain inhibition (Rio et al. 2015). Once the pain is under control, progression to eccentric exercise can be started using the load tolerance principle (Malliaras et al. 2015). Eccentric exercise has been a cornerstone in PT rehabilitation since the 1980s (Steunebrink et al. 2013) and it is a popular (Saithna et al. 2012), extensively investigated therapeutic treatment for PT (Murtaugh & Ihm 2013) that improves functionality (Sosa et al. 2014).

Body weight and body mass index are modifiable intrinsic risk factors for PT. The connection between body composition and the risk for PT is multifaceted and not simply related to loading of the tendon, thus outlining a complexity of confounding interconnected factors (Malliaras & O’Neill 2017). Excessive body weight is responsible for an amplified patellar tendon load (Malliaras & O’Neill 2017) and these combined factors affect microvascularity, which is directly linked to the development of PT (Murtaugh & Ihm 2013).

Irrespective of the type of sport or activity that athletes or the general population participate in, musculoskeletal injuries are a reality (Saragiotto, Di Pierro & Lopes 2013). Repetitive mechanical overload, an extrinsic risk factor (Rowan & Drouin 2013), influences the patellar tendon in surpassing its reparative capacity (Rosso et al. 2015; Rutland et al. 2010), resulting in tendon failure and the development of PT (Malliaras & O’Neill 2017). These risk factors for PT decrease the patellar tendon’s ability to tolerate load over time, which is the typical nature of PT onset (Rosso et al. 2015; Rutland et al. 2010). This, again, highlights the direct connection between the development of PT and the load on the tendon which can be modified with a precise approach in rehabilitation (Vetrano et al. 2013).

Jumping sports participation is an important risk factor (De Vries et al. 2015a; Van der Worp et al. 2011a; Van der Worp et al. 2012), although the estimation of the actual prevalence of PT in specific sports that include jumping has not been identified. This is because of athletes continuing with sport participation even though they experience mild to moderate symptoms. Nonetheless, there is clear evidence that the prevalence of PT in jumping sports is alarmingly high because of the nature of this activity (Schwartz et al. 2015). Vertical jumping overloads the patellar tendon and seems to be because of the velocity and acceleration of ankle dorsiflexion and acceleration of knee flexion (Janssen, Steele & Brown 2015).

The prevalence of PT fluctuates according to the specific jumping sport and between elite and recreational athletes (Rudavsky & Cook 2014). A potential explanation for the prevalence of PT in elite athletes is that they are exposed to several loading and unloading time frames of the patellar tendon because of seasonal breaks and injuries over the years. This progressively decreases the capacity of the patellar tendon to tolerate load, and it becomes vulnerable with any overloading of the tendon, even with simple and minor adaptations in the training regimen (Rudavsky & Cook 2014). On the other hand, one of the main reasons why recreational athletes sustain PT is rapid overloading of the patellar tendon. This overwhelms the tendon and also its capacity to recover, resulting from amplified physical activity, frequency or intensity and the use of incorrect equipment (Torres et al. 2013).

Unfortunately, PT is also prevalent in the general ageing population because of changes in the patellar tendon anatomical structure (Longo et al. 2011), and a quick run across the road or a day gardening may be adequate to overload the patellar tendon and symptoms will appear (Malliaras & O’Neill 2017). This agrees with the findings of age as a non-modifiable intrinsic risk factor for PT.

**FIGURE 2:**
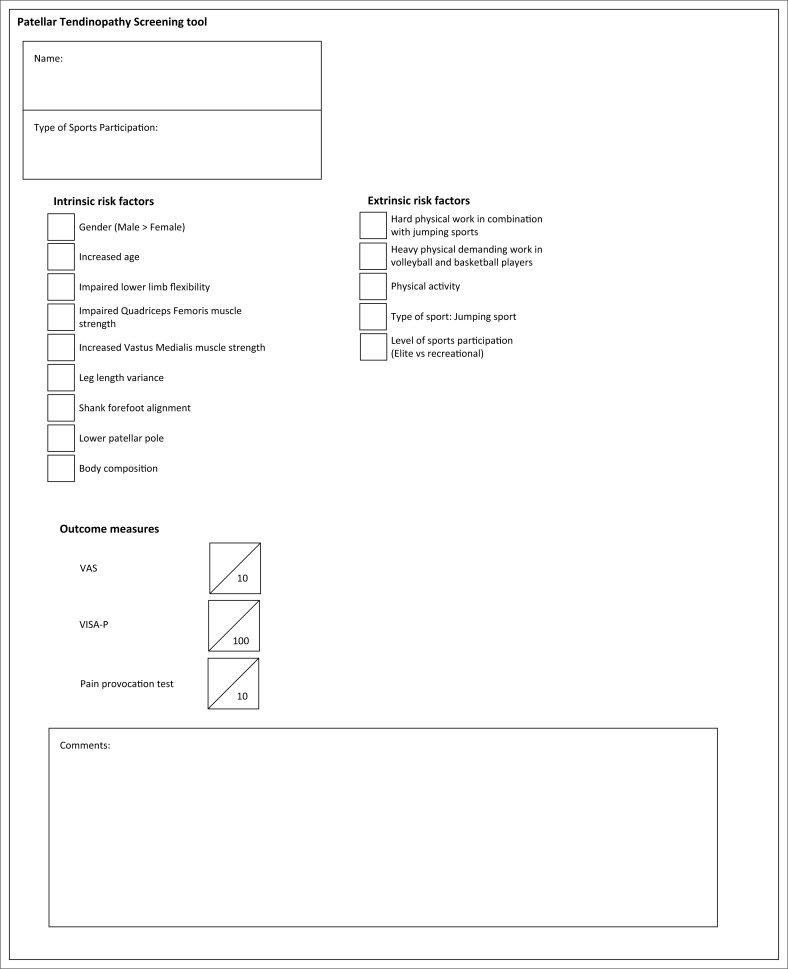
Proposed Patellar Tendinopathy Screening tool.

The secondary aim of the study was to initiate the development of a tool to screen for risk factors, as well as to potentially serve as an outcome measure in the rehabilitation of PT. This may contribute to the refining of PT management in terms of risk factor identification and determining and monitoring pain and function over time. A further possibility of the proposed screening tool in PT (Figure 2) is to potentially increase the awareness of further PT research in the healthcare community, particularly the development of validated PT screening tools.

The identified non-modifiable and modifiable intrinsic and extrinsic risk factors in this systematic review (despite the small sample size of the included articles) are all potentially relevant to the pathology and were used as the basis for the proposed PT screening tool (Figure 2). To broaden the tool to become an outcome measure (Scott et al. 2013), it is suggested that the proposed PT screening tool is combined with two subjective outcome measures and a pain provocation test for load tolerance of the patellar tendon. According to Vetrano et al. (2013), the Victorian Institute of Sports Assessment for Patellar Tendinopathy questionnaire (VISA-P) and Visual Analog Scale (VAS) are well-known subjective questionnaires used extensively in PT rehabilitation. Independently, these outcome measurements have good validity and reliability for application in many clinical settings, including PT rehabilitation (De Vries et al. 2013; Gosens et al. 2012; Hernandez-Sanchez et al. 2017; Malliaras et al. 2015). The VAS is a sensitive, subjective one-dimensional questionnaire that estimates pain intensity in pathology (Hawker et al. 2011). It can measure the athlete’s initial pain experience, pain during rehabilitation and offers precise indications of pain intensity and variations in pain (Vetrano et al. 2013). Patellar tendinopathy symptoms are difficult to quantify, and the VISA-P questionnaire is a disease-specific published clinical scale validated for PT, which contributes to its functional assessment (Vetrano et al. 2013).

Inclusion of a pain provocation test (Malliaras et al. 2015) may add considerable value to the evaluation of symptoms on a daily basis (Rudavsky & Cook 2014), which is helpful in rehabilitation. Determining pain response in PT in terms of aggravation of symptoms at baseline, and during and after loading of energy-storage activities, is a major aspect in the rehabilitation and management of PT pathology in order to make load adaptations. This can be achieved by a 24-h pain provocation test (Malliaras et al. 2015), which consists of a functional assessment test (single-leg decline squat test) (Rudavsky & Cook 2014). If the pain on the provocation test returns to baseline in a 24-h period, it implies that the patellar tendon pain is ‘stable’ and that the tendon has tolerated the load (Malliaras et al. 2015). This test can be advanced to a maximal vertical jump or triple-hop test during the return-to-sport phase of rehabilitation (De Vries et al. 2015b; Malliaras et al. 2015). According to Malliaras et al. (2015), the load tolerance principle is the single most important part of PT rehabilitation.

According to the authors’ knowledge, there are no other PT screening tools. The proposed PT screening tool may possibly be useful in rehabilitation, as it includes a dual function of outlining likely intrinsic and extrinsic risk factors for the development of PT, the estimation of pain and functional impairments and is not indicated for any specific population. The value of the proposed PT screening tool will only be verified if properly tested in appropriate studies (Bishop 2008; McCreesh & Lewis 2013).

## Strength, limitation and recommendations

The strength of this systematic review is that the intrinsic and extrinsic risk factors for PT have been identified. Risk factor identification promotes the development and implementation of prevention strategies in the management of this condition. The evidence on the risk factors was used to suggest a proposed PT screening tool which will need to be tested in appropriate studies. A limitation of this systematic review was the limited number of included articles and a probable reason might be the explicit exclusion of youth populations. Another limitation was the lack of randomised clinical trials to validate the results.

## Conclusion

Intrinsic and extrinsic risk factors for PT were identified in this systematic review. This evidence, as well as appropriate literature, formed the basis for the formulation of a proposed PT screening tool which will require testing to determine its usefulness.

## Appendix 1

### Exclusion of articles because of unsuitability based on title screening (*n* = 49)

Abat, F., Diesel, W.J., Gelber, P.E., Polidori, F., Monllau, J.C. & Sanchez-ibanez, J.M., 2014, ‘Effectiveness of the intratissue percutaneous electrolysis (EPI) technique and isoinertial eccentric exercise in the treatment of patellar tendinopathy at two years follow-up’, *Muscles, Ligaments and Tendons Journal* 4(2), 188–193.

Abstracts of the International Federation of Associations of Anatomists, 2014, viewed 12 September 2016, from http://www.csas.org.cn/ifaa2014/AANAT.pdf

Benner, R.W., Shelbourne, K.D. & Gray, T., 2016, ‘The degree of knee extension does not affect postoperative stability or subsequent graft tear rate after anterior cruciate ligament reconstruction with patellar tendon autograft’, *American Journal of Sports Medicine* 44 (4), 844–849.

Biernat, R., Trzaskoma, Z., Trzaskoma, L. & Czaprowski, D., 2014, ‘Rehabilitation protocol for patellar tendinopathy applied among 16- to 19-year old volleyball players’, *Journal of Strength and Conditioning Research* 28(1), 43–52.

Brockmeyer, M., Diehl, N., Schmitt, C., Kohn, D.M. & Lorbach, O., 2015, ‘Results of surgical treatment of chronic patellar tendinosis (jumper’s knee): A systematic review of the literature’, *The Journal of Arthroscopic & Related Surgery* 31(12), 2424–2429.

Camargo, P.R., Alburquerque-Sendin, F. & Salvini, T.F., 2014, ‘Eccentric training as a new approach for rotator cuff tendinopathy: Review and perspectives’, *World Journal of Orthopedics* 5(5), 634–644.

Campbell, R.S.D. & Dunn, A.J., 2012, ‘Radiological interventions for soft tissue injuries in sport’, *British Journal of Radiology* 85(1016), 1186–1193.

Clark, N.T.M., Bourque, R.D., Schilling, J. & Mckeon, P., 2014, ‘Treatment of patello-femoral pain syndrome in a track athlete’, *International Journal of Athletic Therapy & Training* 19(1), 27–31.

Ellenbecker, T.S., Pieczynski, T.E. & Davies, G.J., 2010, ‘Rehabilitation of the elbow following sports injury’, *Clinics in Sports Medicine* 29(1), 33.

Filardo, G., Kon, E., Di Matteo, B., Di Martino, A., Tesei, G., Pelotti, P. et al., 2014, ‘Platelet-rich plasma injections for the treatment of refractory Achilles tendinopathy: Results at 4 years’, *Blood Transfusion* 12(4), 533–540.

Filardo, G., Kon, E., Di Matteo, B., Pelotti, P., Di Martino, A. & Marcacci, M., 2013, ‘Platelet-rich plasma for the treatment of patellar tendinopathy: Clinical and imaging findings at medium-term follow-up’, *International Orthopaedics* 37(8), 1583–1589.

Gill, T.J., Carroll, K.M. & Hariri, S., 2013, ‘Open patellar tendon debridement for treatment of recalcitrant patellar tendinopathy: Indications, technique, and clinical outcomes after a 2-year minimum follow-up’ *Sports Health: A Multidisciplinary Approach* 5(3), 276.

Hadzic, V., Sattler, T., Markovic, G., Veselko, M. & Dervisevic, E., 2010, ‘The isokinetic strength profile of quadriceps and hamstrings in elite volleyball players’, *Isokinetics and Exercise Science* 18(1), 31–37.

Hall, R., Barber Foss, K., Hewett, T.E. & Myer, G.D., 2015, ‘Sport specialization’s association with an increased risk of developing anterior knee pain in adolescent female athletes’, *Journal of Sport Rehabilitation* 24(1), 31–35.

Hall, M.M. & Rajasekaran, S., 2016, ‘Ultrasound-guided scraping for chronic patellar tendinopathy: A case presentation’, *Journal of Injury, Function, and Rehabilitation* 8(6), 593–596.

Hernandez, V.P., Varela, S.M. & Moraleda, B.R., 2011, ‘Proposal for functional recovery from ruptured anterior cruciate ligament in soccer’ *Revista Internacional de Medicina y Ciencias de la Actividad Fisica y del Deporte* 11(43), 573–591.

Goom, T.S., Malliaras, P., Reiman, M.P. & Purdam, C.R., 2016, ‘Proximal hamstring tendinopathy: Clinical aspects of assessment and management’, *Journal of Orthopaedic & Sports Physical Therap*y 46(6), 483.

Kaux, J.F., Bruyere, O., Croisier, J.L., Forthomme, B., Le Goff, C. & Crielaard, J.M., 2015, ‘One-year follow-up of platelet-rich plasma infiltration to treat chronic proximal patellar tendinopathies’, *Acta Orthopaedica Belgica* 81(2) 251–256.

Kaux, J.F., Croisier, J.L., Bruyère, O., Rodriguez, C., Daniel, C. & Godon, B., 2013, ‘Platelet-rich plasma (PRP) to treat chronic upper patellar tendinopathies’, *British Journal of Sports Medicine* 47(10), 6.

Kaux, J.F., Croisier, J.L., Bruyère, O., Rodriguez De La Cruz, C., Forthomme, B., Brabant, G. et al., 2015, ‘One injection of platelet-rich plasma associated to a submaximal eccentric protocol to treat chronic jumper’s knee’, *The Journal of Sports Medicine and Physical Fitness* 55(9), 953–961.

Kaux, J.F., Croisier, J.L., Forthomme, B., Le Goff, C., Delcour, S., Gothot, A. & Crielaard, J.M., 2015, ‘Exploring the effect of a second closely-timed infiltration of platelet-rich plasma to treat jumper’s knees’, *Annals of Physical & Rehabilitation Medicine* 58, 68.

Kaux, J.-F., Forthomme, B., Namurois, M.-H., Bauvir, P., Defawe, N., Delvaux, F. et al., 2014, ‘Description of a standardized rehabilitation program based on sub-maximal eccentric following a platelet-rich plasma infiltration for jumper’s knee’, *Muscles, Ligaments & Tendons Journal* 4(1), 85–89.

Kim, K.H. & Kim, T.G., 2011, ‘Effects of patellar tendinopathy on the marche-fente movement of female fencing fleuret players’, *Korean Journal of Sport Science* 22(2), 1875–1883.

Kulig, K., Noceti-Dewit, L.M., Reischl, S.F. & Landel, R.F., 2015, ‘Physical therapists’ role in prevention and management of patellar tendinopathy injuries in youth, collegiate, and middle-aged indoor volleyball athletes’, *Brazilian Journal of Physical Therapy* 19(5), 410–420.

Liddle, A.D. & Rodríguez-Merchán, E.C., 2015, ‘Platelet-rich plasma in the treatment of patellar tendinopathy’, *American Journal of Sports Medicine* 43(10), 2583–2590.

Maffulli, N., Giai Via, A. & Oliva, F., 2017, ‘Revision surgery for failed patellar tendinopathy exploration’ *Sports Medicine and Arthroscopy Review* 25(1), 36–40.

Maier, D., Bornebusch, L., Salzmann, G.M., Sudkamp, N.P. & Ogon, P., 2013, ‘Mid- and long-term efficacy of the arthroscopic patellar release for treatment of patellar tendinopathy unresponsive to nonoperative management’, *Arthroscopy-The Journal of Arthroscopic and Related Surgery* 28(8), 1338–1345.

Marcheggiani, M., Giulio, M., Zaffagnini, S., Tsapralis, K., Alessandrini, E., Bonanzinga, T. et al., 2013, ‘Open versus arthroscopic surgical treatment of chronic proximal patellar tendinopathy. A systematic review’, *Knee Surgery, Sports Traumatology, Arthroscopy* 21(2), 351–357.

Martínez-Rodríguez, A., Moreno-Pérez, V., Roche, E. & Vicente-Salar, N., 2013, ‘Planificación dietética y rehabilitación a Largo Plazo de Jugadores profesionales de tenis y fútbol mediante una aproximación multidisciplinar’, *European Journal of Human Movement* 31, 77.

Morton, S., Otto, C., King, J., Perry, D., Crisp, T., Maffulli, N. et al., 2014, ‘High volume image-guided injections for patellar tendinopathy: A combined retrospective and prospective case series’, *Muscles, Ligaments & Tendons Journal* 4(2), 214–219.

Muneta, T., Koga, H., JU, Y.J., Mochizuki, T. & Sekiya, I., 2012, ‘Hyaluronan injection therapy for athletic patients with patellar tendinopathy’, *Journal of Orthopaedic Science* 17(4), 425–431.

Nanos, K.N. & Malanga, G.A., 2015, ‘Treatment of patellar tendinopathy refractory to surgical management using percutaneous ultrasonic tenotomy and platelet-rich plasma injection: A case presentation’, *Journal of Injury, Function, And Rehabilitation* 7(12), 1300–1305.

Ogon, P., Izadpanah, K., Eberbach, H., Lang, G., Südkamp, N.P. & Maier, D., 2017, ‘Prognostic value of MRI in arthroscopic treatment of chronic patellar tendinopathy: A prospective cohort study’, *BioMed Central Musculoskeletal Disorders* 18, 1–9.

Park, B.H., Seo, J.H., Ko, M.H. & Park, S.H., 2013, ‘Reliability and validity of the Korean version VISA-P Questionnaire for patellar tendinopathy in adolescent elite volleyball athletes’, *Annals of Rehabilitation Medicine* 37(5), 698–705.

Pascarella, A., Alam, M., Pascarella, F., Latte, C., Di Salvatore, M.G. & Maffulli, N., 2011, ‘Arthroscopic management of chronic patellar tendinopathy’, *American Journal of Sports Medicine* 39(9), 1975–1983.

Physical Therapy: *News from the Foundation for Physical Therapy 2010*, viewed 12 September 2016, from http://ptjournal.apta.org/content/90/11/1697.full

Plinsinga, M.L., Brink, M.S., Vicenzino, B. & Van Wilgen, C.P., 2015, ‘Evidence of nervous system sensitization in commonly presenting and.persistent painful tendinopathies: A systematic review’, *Journal of Orthopaedic & Sports Physical Therapy* 45(11), 864–875.

Research Report Abstracts, *Physiotherapy 2011*, viewed 12 September 2016, from http://www.physiotherapyjournal.com/issue/S0031-9406(11)X0004-4

Rio, E., Kidgell, D., Moseley, G.L. & Cook, J., 2016, ‘Elevated corticospinal excitability in patellar tendinopathy compared with other anterior knee pain or no pain’, *Scandinavian Journal of Medicine & Science in Sports* 26(9), 1072–1079.

Ryan, M., Wong, A., Rabago, D., Lee, K. & Taunton, J., 2011, ‘Ultrasound-guided injections of hyperosmolar dextrose for overuse patellar tendinopathy: A pilot study’, *British Journal of Sports Medicine* 45(12), 972–977.

Smith, J. & Sellon, J.L., 2014, ‘Comparing PRP injections with ESWT for athletes with chronic patellar tendinopathy’, *Clinical Journal of Sport Medicine: Official Journal of the Canadian Academy of Sport Medicine* 24(1), 88–89.

Stuhlman, C.R., Stowers, K., Stowers, L. & Smith, J., 2016, ‘Current concepts and the role of surgery in the treatment of jumper’s knee’ (English), *Orthopedics* 39(6), e1028–e1035.

Sunding, K., Willberg, L., Werner, S., Alfredson, H., Forssblad, M. & Fahlström, M., 2015, ‘Sclerosing injections and ultrasound-guided arthroscopic shaving for patellar tendinopathy: Good clinical results and decreased tendon thickness after surgery-a medium-term follow-up study’, *Knee Surgery, Sports Traumatology, Arthroscopy* 23(8), 2259–2268.

Van Ark, M., Van Den Akker-Scheek, I., Meijer, L.T.B. & Zwerver, J., 2013, ‘An exercise-based physical therapy program for patients with patellar tendinopathy after platelet-rich plasma injection’ *Physical Therapy in Sport* 14(2), 124–130.

Van Duren, B., Pandit, H., Murray, D. & Gill, H., 2015, ‘Approximation of the functional kinematics of posterior stabilised total knee replacements using a two-dimensional sagittal plane patello-femoral model: Comparing model approximation to in vivo measurement’, *Computer Methods in Biomechanics & Biomedical Engineering* 18(11), 1191–1199.

Verrall, G., Schofield, S. & Brustad, T., 2011, ‘Chronic Achilles tendinopathy treated with eccentric stretching program’, *Foot & Ankle International* 32(9), 843–849.

Willberg, L., Sunding, K., Forssblad, M., Fahlström, M. & Alfredson, H., 2011, ‘Sclerosing polidocanol injections or arthroscopic shaving to treat patellar tendinopathy/jumper’s knee? A randomised controlled study’, *British Journal of Sports Medicine* 45(5), 411–415.

Xu, H., Bloswick, D. & Merryweather, A., 2015, ‘An improved OpenSim gait model with multiple degrees of freedom knee joint and knee ligaments’, *Computer Methods in Biomechanics & Biomedical Engineering* 18(11), 1217–1224.

### Exclusion of articles because of unsuitability based on abstract screening (*n* = 21)

Bond, R.P. & Snyckers, C.H., 2010. ‘Management of sports overuse injuries of the lower limb: An evidence-based review of the literature’, *South African Orthopaedic Journal* 9(2), 48–58.

Caquot, J., Perrochon, A., Bugeaud, J.L., Dumélié, X., Duzou, F. & Daviet, J.C., 2016, ‘Cognitive representations on patellar tendinopathy at the basketball players of the French training centers’, *Annals of Physical & Rehabilitation Medicine* 59, e16.

Caquot, J., Perrochon, A., Bugeaud, J.L., Dumélié, X., Pajon, M. & Daviet, J.C., 2016, ‘Prevalence of pain below patella in the basketball players of the championship of France hope pro A’, *Annals of Physical & Rehabilitation Medicine* 59, e15–e16.

de Carlo, M. & Armstrong, B., 2010, ‘Rehabilitation of the knee following sports injury’, *Clinics in Sports Medicine* 29(1), 81–106.

Dimnjaković, D., Bojanić, I., Smoljanović, T., Mahnik, A. & Barbarić-Peraić, N., 2012, ‘Eccentric exercises in the treatment of overuse injuries of the musculoskeletal system’, *Lijecnic̆kĭ Vjesnik* 134(1–2), 29–41.

Eerkes, K., 2012, ‘Volleyball Injuries’, *Current Sports Medicine Reports* 11(5), 251–256.

Giombini, A., Dragoni, S., Di Cesare, A., Di Cesare, M., Del Buono, A. & Maffulli, N., 2013, ‘Asymptomatic achilles, patellar, and quadriceps tendinopathy: A longitudinal clinical and ultrasonographic study in elite fencers’, *Scandinavian Journal of Medicine & Science in Sports* 23(3), 311–316.

Gómez-Valero, S., García-Pérez, F., Flórez-García, M.T. & Miangolarra-Page, J.C., 2017, ‘A systematic review of self-administered questionnaires for the functional assessment of patients with knee disabilities adapted into Spanish’, *Revista Espanola De Cirugia Ortopedica Y Traumatologia* 61(2), 96–103.

Gordon, A.I., Distefano, L.J., Denegar, C.R., Ragle, R.B., Norman, J.R. & Cheatham, S., 2014, ‘College and professional women’s basketball players’ lower extremity injuries: A survey of career incidence’, *International Journal of Athletic Therapy & Training* 19(5), 25–33.

Hernandez-Sanchez, S., Hidalgo, M.D. & Gomez, A., 2011, ‘Cross-cultural adaptation of VISA-P Score for patellar tendinopathy in Spanish population’, *Journal of Orthopaedic & Sports Physical Therapy* 41(8), 581–591.

Kristensen, J. & Franklyn-Miller, A., 2012, ‘Resistance training in musculoskeletal rehabilitation: A systematic review’, *British Journal of Sports Medicine* 46(10), 719–726.

Moura, D.L., Marques, J.P., Lucas, F.M. & Fonseca, F.P., 2016, ‘Simultaneous bilateral patellar tendon rupture’, *Revista Brasileira De Ortopedia* 52(1), 111–114.

Pas, H.I.M.F.L., Moen, M.H., Haisma, H.J. & Winters, M., 2017, ‘No evidence for the use of stem cell therapy for tendon disorders: A systematic review’, *British Journal of Sports Medicine* 51(13), 996–1002.

Plinsinga, M., Vuvan, V., Stephenson, A., Mellor, R., Heales, L., Van Wilgen, P. et al., 2017, ‘Pain and psychological characteristics in patellar tendinopathy’, *Journal of Science & Medicine in Sport* 20, e20.

Rio, E., Kidgell, D., Moseley, G.L. & Cook, J., 2015, ‘Elevated corticospinal excitability in patellar tendinopathy compared with other anterior knee pain or no pain’, *Scandinavian Journal of Medicine & Science in Sports* 26(9), 1072–1079.

Rio, E., Van Ark, M., Docking, S., Moseley, G.L., Kidgell, D., Gaida, J.E. et al., 2017, ‘Isometric contractions are more analgesic than isotonic contractions for patellar tendon pain: An in-season randomized clinical trial’,. *Clinical Journal of Sport Medicine* 27(3), 253–259.

Schwartz, A., Watson, J.N. & Hutchinson, M.R., 2015, ‘Patellar Tendinopathy’, *Sports Health* 7(5), 415–420.

Wearing, S.C., Locke, S., Smeathers, J.E. & Hooper, S.L., 2015, Tendinopathy alters cumulative transverse strain in the patellar tendon after exercise’, *Medicine & Science in Sports & Exercise* 47(2), 264–271.

White, T. & Clapis, P., 2010, ‘Patellar tendonitis (jumper’s knee) rehabilitation exercises’, *Computerized Registration System – Sports Medicine Advisor* 1, viewed 20 August 2018, from, http://sportmed.com/wp-content/uploads/Combined_Patellar_Tendinitis.pdf

Young, M.A., Cook, J.L., Purdam, C.R., Kiss, Z.S. & Alfredson, H., 2010, ‘Patellar tendon injury (jumper’s knee) rehabilitation exercises: References’, *Computerized Registration System – Sports Medicine Advisor* 1, viewed *20 August 2018,* from *sportmed.com/wp -content/uploads/ Combined_Patellar_Tendinitis.pdf .*

Zhang, Z.J., Ng, G.Y.F., Lee, W.C. & Fu, S.N., 2017, ‘Increase in passive muscle tension of the quadriceps muscle heads in jumping athletes with patellar tendinopathy’, *Scandinavian Journal of Medicine & Science in Sports* 10, 1099–1104.

### Exclusion of articles because of unsuitability based on full-text screening (*n* = 40)

Couppé, C., Kongsgaard, M., Aagaard, P., Vinther, A., Boesen, M., Kjær, M. et al., 2013, ‘Differences in tendon properties in elite badminton players with or without patellar tendinopathy’, *Scandinavian Journal of Medicine & Science in Sports* 23(2), 89–95.

Culvenor, A.G., Cook, J.L., Warden, S.J. & Crossley, K.M., 2011, ‘Infrapatellar fat pad size, but not patellar alignment, is associated with patellar tendinopathy’, *Scandinavian Journal of Medicine & Science in Sports* 21(6), 405–411.

Da Cunha, R.A., Dias, A.N., Santos, M.B. & Lopes, A.D., 2012, ‘Comparative study of two protocols of eccentric exercise on knee pain and function in athletes with patellar tendinopathy: Randomised controlled study’, *Revista Brasileira de Medicina do Esporte* 18(3), 167–170.

De Vries, A., Zwerver, J., Diercks, R., Tak, I., Van Berkel, A., Van Cingel, R. et al., 2015b, ‘Effect of patellar strap and sports tape on pain in patellar tendinopathy: A randomised controlled trial’, *Scandinavian Journal of Medicine and Science in Sports* 26(10), 1217–1224.

Dimitrios, S., 2015, ‘There is lack of evidence to support the effectiveness of therapeutic ultrasound in the management of patellar tendinopathy’, *Physical Therapy Reviews* 20(4), 268–269.

Dimitrios, S., Pantelis, M. & Kalliopi, S., 2012, ‘Comparing the effects of eccentric training with eccentric training and static stretching exercises in the treatment of patellar tendinopathy. A controlled clinical trial’ *Clinical Rehabilitation* 26(5), 423–430.

Edouard, P., Cugy, E., Dolin, R., Morel, N. & Steffen, K., 2016, ‘An injury prevention program is able to reduce the number of injury complaints at medium-term in athletics’, *Annals of Physical & Rehabilitation Medicine* 59, e17–e18.

Frizziero, A., Trainito, S., Oliva, F., Aldini, N.N., Masiero, S. & Maffulli, N., 2014, ‘The role of eccentric exercise in sport rehabilitation’, *British Medical Bulletin* 110(1), 47–75.

Goldman, R.B. & Lentz, T.A., 2010, ‘The use of eccentric overloading exercise for the treatment of patellar tendinosis in an Olympic-style weightlifter: A case report’, *Orthopaedic Practice* 22(2:10), 76–82.

Ho, K.Y. & Kulig, K., 2016, ‘Changes in water content in response to an acute bout of eccentric loading in a patellar tendon with a history of tendinopathy: A case report’, *Physiotherapy Theory & Practice* 32(7), 566–570.

Jain, N., Kemp, S., Hayward, P. & Murray, D.J., 2012, ‘Effect of pre-season screening for patella tendinopathy: The findings of a professional football club’, *Muscles, Ligaments & Tendons Journal* 10.

James, L., Kelly, V. & Beckman, E., 2014, ‘Injury risk management plan for volleyball athletes’, *Sports Medicine* 44(9), 1185–1195.

Kulig, K., Noceti-Dewit, L.M., Reischl, S.F. & Landel, R.F., 2015, ‘Physical therapists’ role in prevention and management of patellar tendinopathy injuries in youth, collegiate, and middle-aged indoor volleyball athletes’, *Brazilian Journal of Physical Therapy* 19(5), 410–420.

Leal, C., Ramon, S., Furia, J., Fernandez, A., Romero, L. & Hernandez-Sierra, L., 2015, ‘Current concepts of shockwave therapy in chronic patellar tendinopathy’, *International Journal of Surgery* 24(B), 160–164.

Lee, W., Zhang, Z., Masci, L., Ng, G., Fu, S., Lee, W.C. et al.,2017, ‘Alterations in mechanical properties of the patellar tendon is associated with pain in athletes with patellar tendinopathy’, *European Journal of Applied Physiology* 117(5), 1039-1045.

Leporace, G., Pereira, G.R., Carmo, R.C.R., Silva, A.C., Cabral, R.P., Silva, N. et al., 2010, ‘Specificity of the myoelectrical activity on the eccentric decline squat at 25 degrees and standard squat with different overloads’, *Revista Brasileira de Medicina do Esporte* 16(3), 205–209.

Malliaras, P., Barton, C.J., Reeves, N.D. & Langberg, H., 2013, ‘Achilles and patellar tendinopathy loading programmes: A systematic review comparing clinical outcomes and identifying potential mechanisms for effectiveness’, *Sports Medicine* 43(4), 267–286.

Malliaras, P., Cook, J., Purdam, C. & Rio, E., 2015, ‘Patellar tendinopathy: Clinical diagnosis, load management, and advice for challenging case presentations’, *Journal of Orthopaedic and Sports Physical Therapy* 45(11), 887–898.

Mani-Babu, S., Morrissey, D., Waugh, C., Screen, H. & Barton, C., 2015, ‘The effectiveness of extracorporeal shock wave therapy in lower limb tendinopathy: A systematic review’, *American Journal of Sports Medicine* 43(3), 752–761.

Mccreesh, K.M., Riley, S.J. & Crotty, J.M., 2013, ‘Neovascularity in patellar tendinopathy and the response to eccentric training: A case report using Power Doppler ultrasound’, *Manual Therapy* 18(6), 602–605.

Mendonça, L.D., Bittencourt, N.F.N., Santos, T.R.T., Silva, A.A. & Fonseca, S.T., 2011, ‘Correlation of age, sex, body mass index and sports modality to patellar rotation in jumping athletes’, *British Journal of Sports Medicine* 45(4), 344.

Morton S., Morrissey D., Valle X., Chan O., Langberg H. & Malliaras P., 2015, ‘Equivalence of online and clinical administration of a patella tendinopathy risk factor and severity questionnaire’, *Scandinavian Journal of Medicine & Science in Sports* 25(5), 670–677.

Morton, S., Williams, S., Valle, X., Diaz-Cueli, D., Malliaras, P. & Morrissey, D., 2017, ‘Patellar tendinopathy and potential risk factors: An international database of cases and controls’, *Clinical Journal of Sport Medicine* 27(5), 468–474.

Rio, E., Kidgell, D., Purdam, C., Gaida, J., Moseley, G.L., Pearce, A.J. & Cook, J., 2015, ‘Isometric exercise induces analgesia and reduces inhibition in patellar tendinopathy’, *British Journal of Sports Medicine* 49(19), 1277–1283.

Romero-Rodriguez, D., Gual, G. & Tesch, P.A., 2011, ‘Efficacy of an inertial resistance training paradigm in the treatment of patellar tendinopathy in athletes: A case-series study’, *Physical Therapy in Sport* 12(1), 43–48.

Saggini, R., Di Stefano, A., Galati, V., Panelli, E., Valeri, M., Di Pancrazio, L. et al., 2012, ‘Long-term effectiveness of combined mechanotransduction treatment in jumper’s knee’, *European Journal of Inflammation* 10(3), 515–524.

Samukawa, M., 2011, ‘Management of patellar tendinosis in a freestyle mogul skier’, *International Journal of Athletic Therapy and Training* 16(2), 12–15.

Scattone, Silva, R., Nakagawa, T.H., Ferreira, A.L.G., Garcia, L.C., Santos, J.E.M. & Serrão, F.V., 2016, ‘Lower limb strength and flexibility in athletes with and without patellar tendinopathy’, *Physical Therapy in Sport* 20, 19.

Silva, R.S., Ferreira, A.L.G., Nakagawa, T.H., Santos, J.M. & Serrão, F.V., 2015, ‘Rehabilitation of patellar tendinopathy using hip extensor strengthening and landing-strategy modification: Case report with 6-month follow-up’, *Journal of Orthopaedic and Sports Physical Therapy* 45(11), 899–909.

Simpson, M. & Smith, T.O., 2011, ‘Quadriceps tendinopathy – A forgotten pathology for physiotherapists? A systematic review of the current evidence-base’, *Physical Therapy Reviews* 16(6), 455–461.

Sorenson, S.C., Arya, S., Souza, R.B., Pollard, C.D., Salem, G.J. & Kulig, K., 2010, ‘Knee extensor dynamics in the volleyball approach jump: The influence of patellar tendinopathy’, *Journal of Orthopaedic & Sports Physical Therapy* 40(9), 568–576.

Souza, R.B., Arya, S., Pollard, C.D., Salem, G. & Kulig, K., 2010, ‘Patellar tendinopathy alters the distribution of lower extremity net joint movements during hopping’, *Journal of Applied Biomechanics* 26(3), 249–255.

Thijs, K.M., Backx, F.J.G., Moen, M.H., Zwerver, J., Steeneken, V., Rayer, S. et al., 2017, ‘Effectiveness of shockwave treatment combined with eccentric training for patellar tendinopathy: A double-blinded randomized study’, *Clinical Journal of Sport Medicine* 27(2), 89–96.

Van Der Worp, H., Zwerver, J., Van Den Akker-Scheek, I. & Diercks, R.L., 2011c, ‘The TOPSHOCK study: Effectiveness of radial shockwave therapy compared to focused shockwave therapy for treating patellar tendinopathy – Design of a randomised controlled trail’, *BMC Musculoskeletal Disorders* 12, 229–234.

Wilgen, C.P., Konopka, K.H., Keizer, D. & Zwerver, J., 2013, ‘Do patients with chronic patellar tendinopathy have an altered somatosensory profile? A quantitative sensory testing (QST) study’, *Scandinavian Journal of Medicine & Science in Sports* 23(2), 149–155.

Yun, S., Jin, W., Park, Y.K., Kim, G., Yoon, S., Park, S. et al., 2015, ‘Increased signal intensity at the proximal patellar tendon: Correlation between MR imaging and histology in eight cadavers and clinical MR imaging studies’, *European Radiology* 25(10), 2976–2983.

Zhang, Z., Ng, G., FU, S., Zhang, Z.J., Ng, G.Y.F. & Fu, S.N., 2015, ‘Effects of habitual loading on patellar tendon mechanical and morphological properties in basketball and volleyball players’, *European Journal of Applied Physiology* 115(11), 2263–2269.

Zhang, Z.J., Ng, G.Y., Lee, W.C. & Fu, S.N., 2014, ‘Changes in morphological and elastic properties of patellar tendon in athletes with unilateral patellar tendinopathy and their relationships with pain and functional disability’, *PLoS One* 9(10), 1–9.

Zwerver, J., Dekker, F. & Pepping, G.J., 2010, ‘Patient guided Piezo-electric Extracorporeal Shockwave Therapy as treatment for chronic severe patellar tendinopathy: A pilot study’, *Journal of Back and Musculoskeletal Rehabilitation* 23(3), 111–115.

### Exclusion of articles because of poor methodological quality (*n* = 7)

Frizziero, A., Vittadini, F., Fusco, A., Giombini, A. & Masiero, S., 2016, ‘Efficacy of eccentric exercise in lower limb tendinopathies in athletes’, *The Journal of Sports Medicine and Physical Fitness* 56(11), 1352–1358.

Malliaras, P., Cook, J., Purdam, C. & Rio, E. (2015). Patellar Tendinopathy: Clinical Diagnosis, Load Management, and Advice for Challenging Case Presentations. *Journal of Orthopaedic & Sports Physical Therapy* 45(11), 887–898.

Nagraba, L., Mitek, T., Stolarczyk, A., Lebiediew, A. & Przymorski, T., 2010, ‘Tendinopathy of the patellar ligament (jumper’s knee) – Diagnostics and treatment’, *Arthroscopy and Joint Surgery* 6(3–4), 19–23.

Pearson, S.J. & Hussain, S.R., 2014, ‘Region-specific tendon properties and patellar tendinopathy: A wider understanding’, *Sports Medicine* 44(8), 1101–1112.

Rodriguez-Merchan, E.C., 2013, ‘The treatment of patellar tendinopathy’, *Journal of Orthopaedic Traumatology* 14, 77–81.

Rudavsky, A. & Cook, J., 2014, ‘Physiotherapy management of patellar tendinopathy (jumper’s knee)’, *Journal of Physiotherapy* 60(3), 122–129.

Rutland, M., O’Connell, D., Brismee, J.M., Sizer, P., Apte, G. & O’Connell, J., 2010, ‘Evidence-supported rehabilitation of patellar tendinopathy’, *North American Journal of Sports Physical Therapy* 5(3), 166–178.

### Methodology quality scoring of the individual articles

#### Systematic review

**Table:**

Author	AMSTAR Checklist Scoring
Van Der Worp et al. (2011b)	8/11

#### Quantitative research

**Table:**

Author	National Institute for Health and Excellence Checklist Scoring
De Vries et al. (2015a)	21/27
Mendonça et al. (2016)	18/27
Toppi et al. (2015)	16/27
Van der Worp et al. (2011a)	16/27
Van der Worp et al. (2012)	15/27
